# MRI radiomics-based machine learning model integrated with clinic-radiological features for preoperative differentiation of sinonasal inverted papilloma and malignant sinonasal tumors

**DOI:** 10.3389/fonc.2022.1003639

**Published:** 2022-09-23

**Authors:** Jinming Gu, Qiang Yu, Quanjiang Li, Juan Peng, Fajin Lv, Beibei Gong, Xiaodi Zhang

**Affiliations:** ^1^ Department of Radiology, The First Affiliated Hospital of Chongqing Medical University, Chongqing, China; ^2^ Department of Clinical Science, Philips Healthcare, Chengdu, China

**Keywords:** radiomics, machine learning, magnetic resonance imaging, sinonasal inverted papilloma, malignant sinonasal tumor, differential diagnosis

## Abstract

**Objective:**

To explore the best MRI radiomics-based machine learning model for differentiation of sinonasal inverted papilloma (SNIP) and malignant sinonasal tumor (MST), and investigate whether the combination of radiomics features and clinic–radiological features can produce a superior diagnostic performance.

**Methods:**

The database of 247 patients with SNIP (n=106) or MST (n=141) were analyzed. Dataset from scanner A were randomly divided into training set (n=135) and test set 1 (n=58) in a ratio of 7:3, and dataset from scanner B and C were used as an additional independent test set 2 (n=54). Fourteen clinic-radiological features were analyzed by using univariate analysis, and those with significant differences were applied to construct clinical model. Based on the radiomics features extracted from single sequence (T2WI or CE-T1WI) and combined sequence, four commonly used classifiers (logistic regression (LR), support vector machine (SVM), decision tree (DT) and k-nearest neighbor (KNN)) were employed to constitute twelve different machine learning models, and the best-performing one was confirmed as the optimal radiomics model. Furthermore, a combined model incorporated best radiomics feature subsets and clinic-radiological features was developed. The diagnostic performances of these models were assessed by the area under the receiver operating characteristic (ROC) curve (AUC) and the calibration curves.

**Results:**

Five clinic-radiological features (age, convoluted cerebriform pattern sign, heterogeneity, adjacent bone involvement and infiltration of surrounding tissue) were considered to be significantly different between the tumor groups (*P* < 0.05). Among the twelve machine learning models, the T2WI-SVM model exhibited optimal predictive efficacy for classification tasks on the two test sets, with the AUC of 0.878 and 0.914, respectively. For three types of diagnostic models, the combined model achieved highest AUC of 0.912 (95%CI: 0.807-0.970) and 0.927 (95%CI: 0.823-0.980) for differentiation of SNIP and MST in test 1 and test 2 sets, which performed prominently better than clinical model (*P*=0.011, 0.005), but not significantly different from the optimal radiomics model (*P*=0.100, 0.452).

**Conclusion:**

The machine learning model based on T2WI sequence and SVM classifier achieved best performance in differentiation of SNIP and MST, and the combination of radiomics features and clinic-radiological features significantly improved the diagnostic capability of the model.

## Introduction

Sinonasal inverted papilloma (SNIP) is a common benign epithelial sinonasal tumor of Schneiderian mucosa origin (1). Although histologically benign, it is a biologically aggressive tumor characterized by a known propensity for a high rate of local invasiveness, recurrence and a risk of malignant transformation (2), which is fundamentally different from other benign sinonasal tumors. The similar clinical symptoms and overlapping imaging features between SNIP and malignant sinonasal tumor (MST) have often confounded their clinical diagnosis (3, 4), but their prognostic and treatment strategies are quite different. MST are usually detected at an advanced stage and have poor prognosis. Clinically, patients with MST are usually treated with a comprehensive therapeutic strategy of surgery combined with adjuvant radiotherapy and/or chemotherapy. For patients with SNIP, complete surgical resection alone could achieve a good therapeutic outcome (5, 6). Therefore, an accurate preoperative diagnosis of sinonasal tumors is essential to develop appropriate treatment strategies and assess prognosis.

Existing examination methods for sinonasal tumor diagnosis have limitations in accuracy, quantification and objectivity of the results. Sampling errors and limited sampling sometimes reduce the diagnostic sensitivity of endoscopic incisional biopsy, because sinonasal tumors are often accompanied by inflammatory secretions and polyps (7). Traditional radiology diagnosis is subjective, and differentiation of SNIP and MST by analyzing morphological features on conventional CT or MRI images is challenging, because these features are often nonspecific and overlapped (1, 3, 8). Functional magnetic resonance imaging such as diffusion weighted imaging (DWI), apparent diffusion coefficient (ADC) and dynamic contrast-enhanced MRI (DCE-MRI) have been proved to provide additional valuable information about functional and tissue physiological (9, 10). However, magnetic susceptibility artifact caused by the presence of bone and air around the nasal cavity and sinuses can distort the views of DWI image of the region, which may result in measurement bias (3, 11). Accordingly, there is a need to develop a non-invasive and accurate preoperative diagnosis method to complement additional useful information as a decision aid, and help guide endoscopic biopsy for the sinonasal tumors.

Fortunately, radiomics holds the potential to address these barriers, which refers to the high-throughput extraction and subsequent processing of quantitative features from medical images (12). As an objective, non-invasive and repeatable tool, radiomics can be used to characterize intratumor heterogeneity and decode tumor phenotype (13, 14). Recently, Ramkumar et al. (15) found that MRI-based texture analysis had the potential to classify SNIP and squamous cell carcinoma. Another previous study (16) showed that machine learning models based on MRI radiomics and morphological features achieved satisfactory predictive efficiency in differentiating SNIP from SNIP-transformed squamous cell carcinomas. Despite radiomics method has been utilized in prior studies to classify sinonasal tumors (17, 18), and has promising performance, the most appropriate sequence and machine learning classifier for model construction remain undetermined.

Therefore, the purpose of this study was to explore the optimal machine learning model for differentiating between SNIP and MST, and investigate whether the combination of radiomics features and clinic–radiological features can produce a superior diagnostic performance, and validate the generalization performance of the model on an independent test set with different scanners.

## Materials and methods

**Figure 1:**
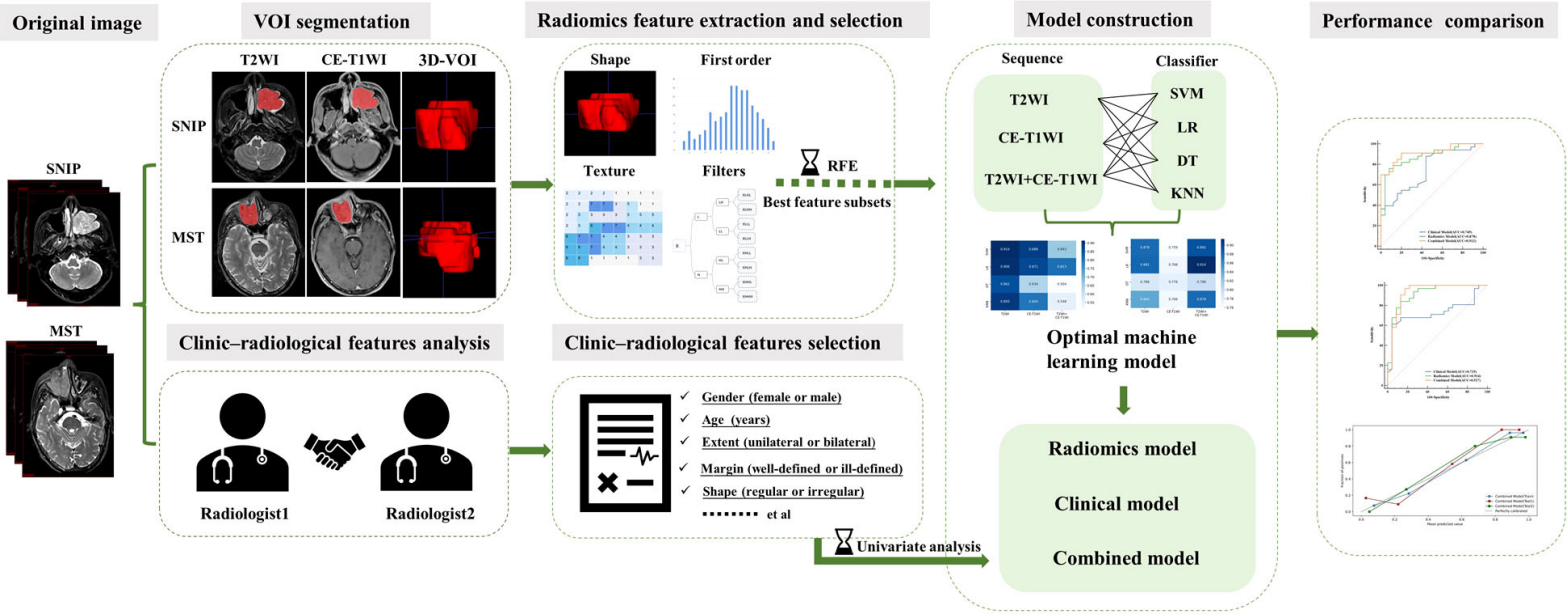
The research workflow of the whole study. SNIP, sinonasal inverted papilloma; MST, malignant sinonasal tumor; 3D-VOI, three-dimensional volume of interest; SVM, support vector machine; LR, logistic regression; DT, decision tree; KNN, k-nearest neighbor.

This retrospective study was approved by the Institutional Review Board of our institution (approval ID: 2020080), and the requirement for the patient informed consent was waived. The research workflow of this study is presented in **Figure 1**.

### Study population

**Figure 2:**
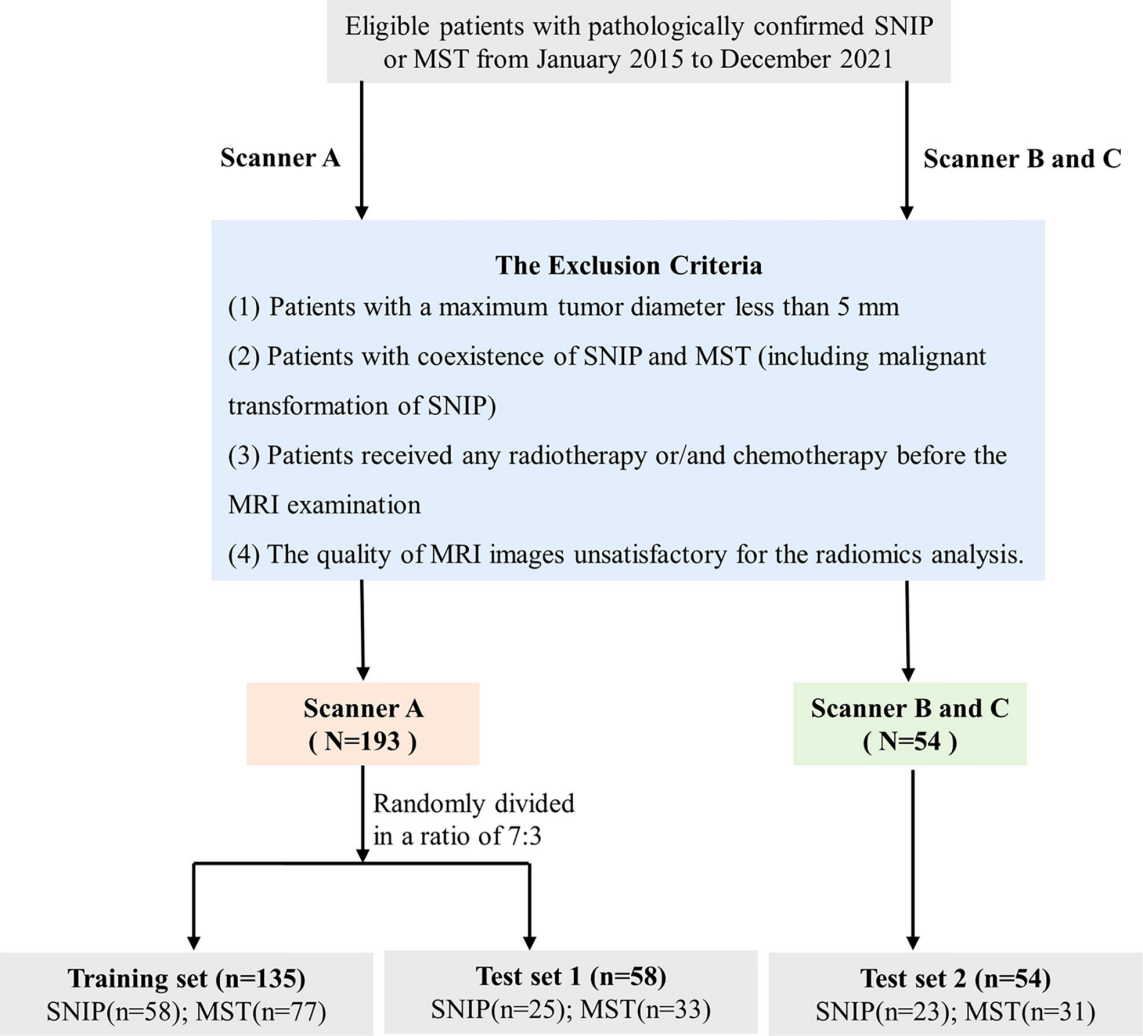
Flowchart of the study population selection. SNIP, sinonasal inverted papilloma; MST, malignant sinonasal tumor; MRI, magnetic resonance imaging.

The database of patients with sinonasal tumors admitted to our institution between January 2015 and December 2021 were consecutively obtained, and all the relevant clinical and radiological data were retrospectively reviewed. All patients had definitive surgically or biopsy confirmed pathological results. The inclusion criteria were as follows: (1) patients with pathologically confirmed SNIP or primary MST; (2) MRI examination was performed within 2 weeks before biopsy or surgery; (3) patients with no previous history of malignant tumors; and (4) patients with complete clinical and image data. The exclusion criteria were as follows: (1) patients with a maximum tumor diameter less than 5 mm; (2) patients with coexistence of SNIP and MST (including malignant transformation of SNIP); (3) patients received any radiotherapy or/and chemotherapy before the MRI examination; and (4) the quality of MRI images unsatisfactory for the radiomics analysis. All tumors were classified as SNIP or MST according to the histopathological results and the latest World Health Organization classification (19). Three most common types of tumors were included in the MST groups, namely squamous cell carcinoma (SCC), lymphoma and sinonasal malignant melanoma (SMM). The enrolment process of patients for this study is illustrated in **Figure 2**.

A total of 247 patients met the criteria were enrolled in this study. Images were collected on three different scanners at our institution. Dataset from scanner A were randomly divided into training set (n=135) and test set 1 (n=58) in a ratio of 7:3, whereas the dataset from scanner B and C were used as an additional independent test set 2 (n=54). Model training and hyperparameters optimization was carried out on the training set, and model performance evaluation was performed on the test set 1. Additionally, the final model was applied in an independent test set 2 with different scanners to obtain an unbiased estimate of model performance.

### MRI image acquisition

Patients from training set and test set 1 were examined on scanner A (Siemens Magnetom Essenza 1.5-T scanner), and patients from test set 2 were examined on scanner B and C (GE Signa HDxt 3.0-T scanner, Siemens Magnetom Skyra 3.0-T scanner). Axial and coronal fast-spin-echo (FSE) T1-weighted image (T1WI), fat-suppressed T2-weighted image (T2WI) and contrast-enhanced T1-weighted image (CE-T1WI) were performed on all patients. Gadopentetate dimeglumine (Gd⁃DTPA) contrast agent was administered intravenously at an injection dose of 0.1 mL/kg body weight and rate of 2.5mL/sec. The scanning protocol and parameters are detailed in the **Supplementary Table S1**.

### Clinic-radiological features analysis

With no knowledge of the clinical-pathological data, two radiologists (with 6 and 12 years of head and neck radiology experience, respectively)independently evaluated the MRI radiological features, and inconsistencies were resolved through consultation until consensus was reached. Fourteen clinic-radiological features were evaluated as follows: (a) gender; (b) age; (c) size; (d) location; (e) extent; (f) shape; (g) margin; (h) convoluted cerebriform pattern sign (CCP sign, defined as alternating hypointense and hyperintense bands on T2-weighted and contrast-enhanced T1-weighted images) (1); (i) T1 high signal; (j) T2 low signal; (k) heterogeneity; (l) necrosis (defined as a non-enhanced area with hypointensity on T1WI and hyperintensity on T2WI) (4); (m) adjacent bone involvement (ABI, including none, bone sclerosis, bone destruction and both); (n) infiltration of surrounding tissue (IST, defined as an extension into the periantral soft tissue, intracranial structures, orbital, cavernous sinus, skin or subcutaneous tissue et al) (4). Cohen’s kappa was used to measure the interrater agreement between the two radiologists’ evaluations of MRI radiological features. Univariate analysis was performed to identify clinic-radiological features with significant differences between SNIP and MST, and these features were applied to build the clinical model. The Chi-square test or Fisher exact test, Student’s *t*-test or Mann–Whitney U-test were used for univariate analysis as appropriate.

### Image segmentation, preprocessing and radiomics feature extraction

**Figure 3:**
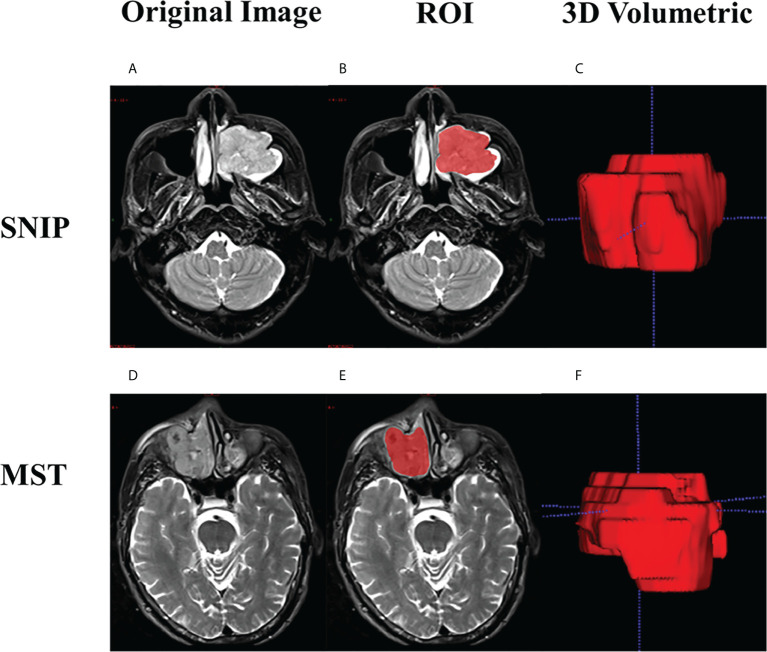
Examples of manual ROI segmentation. Case 1: a 47-year-old man with SNIP in the left maxillary sinus, **(A)** Original image; **(B)** The ROI contour formed by manual segmentation; **(C)** 3D volumetric reconstruction. Case 2: a 60-year-old man with squamous cell carcinoma in the right maxillary sinus, **(D)** Original image; **(E)** The ROI contour formed by manual segmentation; **(F)** 3D volumetric reconstruction. SNIP, sinonasal inverted papilloma; MST, malignant sinonasal tumor; ROI, region of interest; 3D, three-dimensional.

Radiologists performed the three-dimensional (3D) volume of interest (VOI) delineation using ITK-SNAP software (version 3.6.0, http://www.itksnap.org). The two-dimensional (2D) region of interest (ROI) was manually delineated around the outermost boundaries of tumors slice by slice to form the 3D-VOI on axial T2WI and CE-T1WI sequences. The ROI segmentation contained the entire primary tumor, but avoided covering adjacent normal tissue, bone and peripheral inflammation regions. The examples of manual ROI segmentation are presented in **Figure 3**.

In order to reduce the effect of scanning parameters variations, a series of preprocessing methods were performed prior to radiomics feature extraction. All images were resampled to the voxel spacing of 1×1×1 mm³, and the per-image pixel intensity distributions was normalized. Then, 1130 radiomics features were extracted from each VOI by using PyRadiomics version 3.0.1, including shape features, first order statistics and texture features, and we performed different transformations of these features (such as wavelet filters and Laplacian of Gaussian (log) filter). Mathematical definitions and calculation formula for these radiomics features can be found in PyRadiomics package (https://pyradiomics.readthedocs.io/en/latest).

In order to evaluate intra-observer reproducibility and inter-observer reliability of radiomics features, 50 cases of MR images were randomly chosen to calculate the intraclass correlation coefficient (ICC). Radiologist 1 and radiologist 2 performed ROI segmentation independently for the same period, and radiologists 1 performed segmentation again after 2 weeks. Features with ICC above 0.75 were considered to be highly stability and consistency (20). The remaining image segmentation was carried out by radiologist 1.

The Spearman correlation coefficients were used for preliminary screening of radiomics features. In this procedure, feature pairs with Spearman correlation coefficients greater than 0.9 were identified to be highly correlated, and each pair of features only one were remained in the feature set. All features were then normalized by the use of Z-score to reduce the influence of differences in dimensions between different features. Thereafter, recursive feature elimination (RFE) algorithm was implemented to further screen the radiomics features, and ensure retain the best feature subsets. Briefly, after a classifier was trained, the least important features were dropped, and then we trained a new model using those features remaining, repeated this process until the feature set is reduced to a previously selected number of features (21).

### Optimal machine learning model selection

Based on the radiomics features extracted from single sequence (T2WI or CE-T1WI) and combined sequence (the combination of T2WI and CE-T1WI), four frequently used classifiers such as logistic regression (LR), support vector machine (SVM), decision tree (DT) and k-nearest neighbor (KNN) have been employed to constitute twelve different machine learning models. Herein, the classification capabilities of different machine learning models were compared to identify the best performing one. For model training, the GridSearchCV function (10-fold cross-validation grid search) was used to tune and optimize the model hyperparameters in the training set. The prediction performances of all machine learning models were validated in the test 1 and test 2 sets.

### Development and performance assessment of the clinical model, radiomics model and combined model

Based on the optimal classifiers and feature subset selected above, three types of diagnostic models were developed to differentiate SNIP from MST. A clinical model constructed with clinic-radiological features alone, a radiomics model constructed with radiomics features alone, and a combined model constructed with the combination of both. The area under the receiver operating characteristic (ROC) curve (AUC), accuracy, sensitivity, specificity, positive predictive value (PPV) and negative predictive value (NPV) were used to evaluate the prediction performance of each model. The calibration curves were plotted to assess the degree of deviation between the model predictions and actual outcomes. The DeLong test was used to analyze the statistical differences of AUC values between different models.

### Statistical analysis

Quantitative variables were expressed as mean values ± standard deviations or median with interquartile range as appropriate, with differences analyzed using Student’s *t*-test or Mann–Whitney *U*-test, respectively. Categorical variables were expressed as frequencies or percentages, with differences analyzed using the Chi-square test or Fisher exact test as appropriate. Statistical analysis was performed with IBM SPSS Statistics (version 24.0) software, with statistical significance set at 0.05. The machine learning classifiers were implemented by the Python version 3.7.6 “scikit-learn version 1.0.1” package.

## Results

### Demographic information

Eventually, a total of 247 patients were studied, including 106 patients with SNIP and 141 patients with MST (57 squamous cell carcinoma, 39 lymphoma and 45 sinonasal malignant melanoma). The demographic information of training set, test set 1 and test set 2 are summarized in **Table 1**. There were no statistically significant differences in patient age, gender and tumor type between the training set and test set 1, and also no significant differences between the training set and test set 2 in these baseline characteristics.

**Table 1:** Demographic information of the study population.

Characteristic	Training set	Test set 1	Test set 2	*P*1	*P*2
Scanner	Scanner A	Scanner A	Scanner B, C		
Number of patients	135	58	54		
Gender				0.992	0.588
Male	93	40	35		
Female	42	18	19		
Age (years)	62.00(18.00)	58.00(14.25)	59.50(22.25)	0.332	0.713
Tumor type				0.986	0.963
SNIP	58	25	23		
MST	77	33	31		
SCC	29	16	12		
Lymphoma	22	7	10		
SMM	26	10	9		

P_1_ represent the P values of comparison between training set and test set 1. P_2_ represent the P values of comparison between training set and test set 2. SNIP, sinonasal inverted papilloma; MST, malignant sinonasal tumor; SCC, squamous cell carcinoma; SMM, sinonasal malignant melanoma.

### The analysis result of the clinic-radiological features

Interrater agreement between the two radiologists’ assessments of MRI radiological features was good, with the kappa coefficients greater than 0.80 (**Supplementary Table S2**). **Table 2** shows the univariate analysis result of the clinic-radiological features between SNIP and MST groups. In the training, test 1 and test 2 sets, statistically significant differences were found in age, CCP sign, heterogeneity, adjacent bone involvement and infiltration of surrounding tissue between the tumor groups (*P* < 0.05). Therefore, the above five clinic-radiological features were applied to establish the clinical model.

**Table 2:** Analysis result of the clinic–radiological features between SNIP and MST.

**Characteristics**	**Training set (n=135)**			**Test set 1 (n=58)**			**Test set 2 (n=54)**		
	**SNIP (n=58)**	**MST (n=77)**	***P*_1_**	**SNIP (n=25)**	**MST (n=33)**	***P*_2_**	**SNIP** **(n=23)**	**MST** **(n=31)**	***P*_3_**
Gender^*^			0.008			0.664			0.075
Male	47(81.0%)	46(59.7%)		18(72.0%)	22(66.7%)		18(78.3%)	17(54.8%)	
Female	11(19.0%)	31(40.3%)		7(28.0%)	11(33.3%)		5(21.7%)	14(45.2%)	
Age (years)^#^	54.50(20.00)	64.00(14.00)	0.004	52.00(14.00)	61.00(15.00)	0.019	53.00(13.00)	68.00(25.00)	0.021
Size (mm)^#^	34.28(14.35)	39.70(19.88)	0.317	43.15(14.98)	38.65(22.20)	0.583	34.35(23.55)	39.55(24.40)	0.773
Location^*^			0.023			0.465			0.802
Frontal sinus	1(1.7%)	1(1.3%)		0(0%)	1(3.0%)		0(0%)	0(0%)	
Ethmoid sinus	15(25.9%)	7(9.1%)		6(24.0%)	7(21.2%)		4(17.4%)	4(12.9%)	
Sphenoid sinus	2(3.4%)	1(1.3%)		0(0%)	0(0%)		0(0%)	0(0%)	
Maxillary sinus	18(31.0%)	22(28.6%)		13(52.0%)	12(36.4%)		8(34.8%)	9(29.0%)	
Nasal cavity	22(37.9%)	46(59.7%)		6(24.0%)	13(39.4%)		11(47.8%)	18(58.1%)	
Extent^*^			0.350			0.217			1.000
Unilateral	54(93.1%)	68(88.3%)		24(96.0%)	27(81.8%)		22(95.7%)	29(93.5%)	
Bilateral	4(6.9%)	9(11.7%)		1(4.0%)	6(18.2%)		1(4.3%)	2(6.5%)	
Shape^*^			0.494			0.536			0.693
Regular	6(10.3%)	11(14.3%)		1(4.0%)	4(12.1%)		2(8.7%)	5(16.1%)	
Irregular	52(89.7%)	66(85.7%)		24(96.0%)	29(87.9%)		21(91.3%)	26(83.9%)	
Margin^*^			0.228			0.588			0.766
Well-defined	12(20.7%)	23(29.9%)		6(24.0%)	6(18.2%)		6(26.1%)	7(22.6%)	
Ill-defined	46(79.3%)	54(70.1%)		19(76.0%)	27(81.8%)		17(73.9%)	24(77.4%)	
CCP sign^*^	19(32.8%)	8(10.4%)	0.001	12(48.0%)	3(9.1%)	0.001	8(34.8%)	2(6.5%)	0.022
T1 high signal^*^	13(22.4%)	21(27.3%)	0.520	8(32.0%)	9(27.3%)	0.695	1(4.3%)	9(29.0%)	0.051
T2 low signal^*^	9(15.5%)	16(20.8%)	0.436	8(32.0%)	5(15.2%)	0.128	1(4.3%)	6 (19.4%)	0.225
Heterogeneity^*^	55(94.8%)	54(70.1%)	<0.001	24(96.0%)	22(66.7%)	0.006	21(91.3%)	19(61.3%)	0.013
Necrosis^*^	7(12.1%)	18(23.4%)	0.094	3(12.0%)	8(24.2%)	0.401	1(4.3%)	7(22.6%)	0.140
ABI^*^			0.009			0.021			0.010
None	33(56.9%)	41(53.2%)		9(36.0%)	14(42.4%)		14(60.9%)	17(54.8%)	
Bone sclerosis	12(20.7%)	4(5.2%)		9(36.0%)	2(6.1%)		6(26.1%)	2(6.5%)	
Bone destruction	6(10.3%)	21(27.3%)		3(12.0%)	11(33.3%)		1(4.3%)	11(35.5%)	
Both	7(12.1%)	11(14.3%)		4(16.0%)	6(18.2%)		2(8.7%)	1(3.2%)	
IST^*^	12(20.7%)	33(42.9%)	0.007	7(28.0%)	19(57.6%)	0.025	3(13.0%)	14(45.2%)	0.012

#Data are quantitative variables, presented as mean ± standard deviation or median (quartile), p-value was calculated with Student’s t-test or Mann-Whitney U-test. *Data are categorical variables, expressed as frequencies or percentages, p-value was calculated with the χ2 or Fisher exact test. The analysis result of CCP sign, T1 high signal, T2 low signal, heterogeneity, necrosis and IST is registered as absent or present, and present by default in the table. CCP, convoluted cerebriform pattern; ABI, adjacent bone involvement; IST, infiltration of surrounding tissue; SNIP, sinonasal inverted papilloma; MST, malignant sinonasal tumor.

### Radiomics features selection

**Figure 4:**
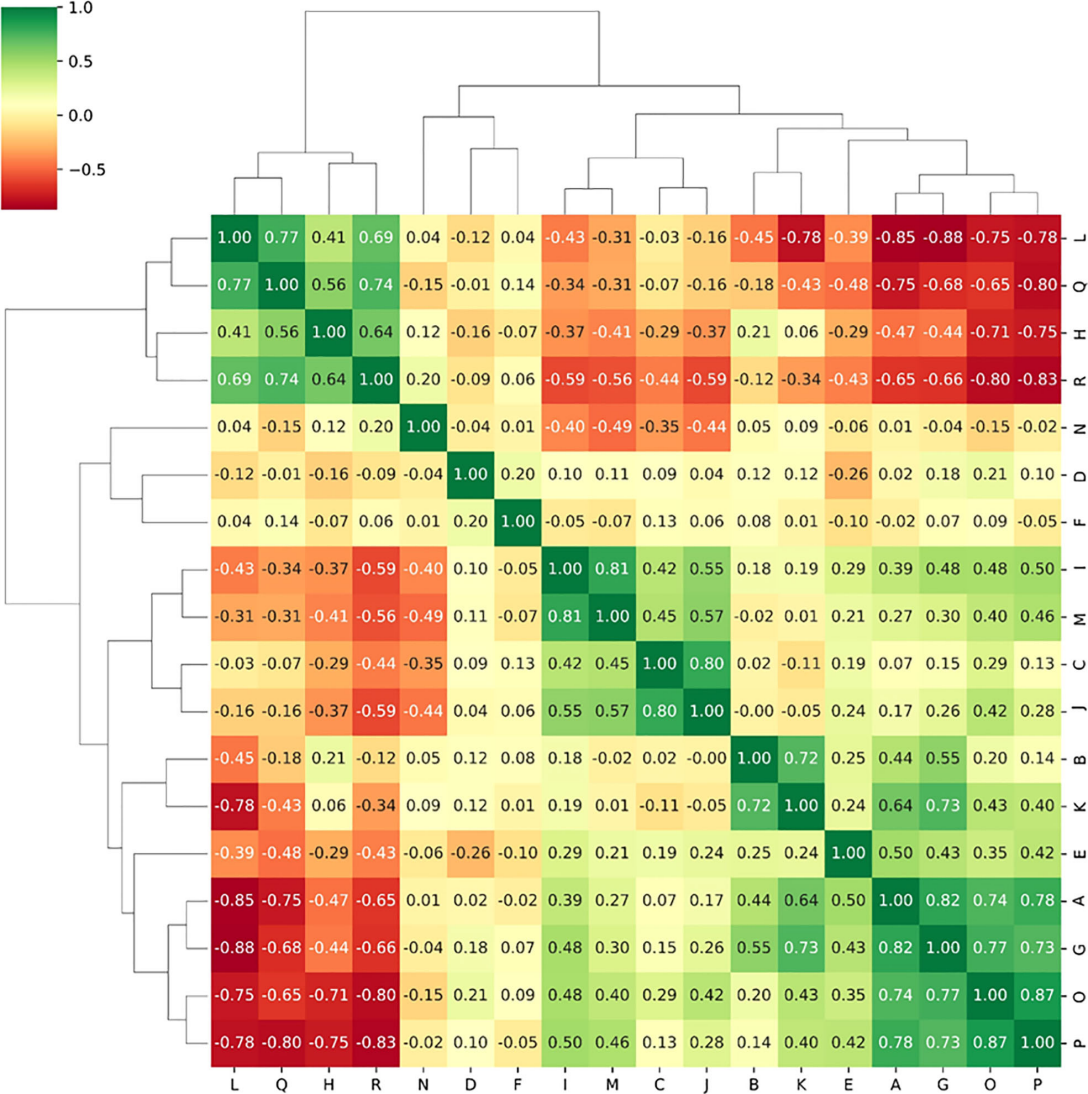
The Spearman correlation coefficients between radiomics features selected from T2WI sequence.

1130 radiomics features were extracted from each sequence, respectively. ICC analysis identified high stability features with ICC greater than 0.75 for subsequent analysis, including 1095 T2WI features and 1101 CE-T1WI features. The Spearman correlation coefficient was used to rejected those similar features with high correlations. After screening using RFE algorithm, 18 radiomics features from each single sequence and combined sequence were reserved for subsequent radiomics model construction, respectively. **Figure 4** showed the Spearman correlation coefficients of 18 radiomics features screened from T2WI sequence, and the variables A to R represent the corresponding radiomics features, which are detailed in **Table 3**.

**Table 3:** Radiomics features selected from T2WI sequence.

Variables	Radiomics features
A	wavelet-LLH_firstorder_10Percentile
B	wavelet-LHL_firstorder_Mean
C	wavelet-LHL_glszm_SmallAreaLowGrayLevelEmphasis
D	wavelet-LHH_glszm_SmallAreaEmphasis
E	wavelet-HHH_glcm_Idm
F	wavelet-LLL_glcm_MCC
G	log-sigma-1-0-mm-3D_firstorder_10Percentile
H	log-sigma-1-0-mm-3D_firstorder_90Percentile
I	log-sigma-2-0-mm-3D_glcm_ClusterShade
J	log-sigma-2-0-mm-3D_glszm_SmallAreaLowGrayLevelEmphasis
K	log-sigma-3-0-mm-3D_firstorder_Median
L	log-sigma-3-0-mm-3D_firstorder_RootMeanSquared
M	log-sigma-3-0-mm-3D_glcm_ClusterShade
N	log-sigma-3-0-mm-3D_glcm_Correlation
O	log-sigma-3-0-mm-3D_glcm_MaximumProbability
P	log-sigma-3-0-mm-3D_glszm_LargeAreaLowGrayLevelEmphasis
Q	log-sigma-3-0-mm-3D_glszm_SmallAreaEmphasis
R	log-sigma-3-0-mm-3D_ngtdm_Complexity

### Predictive efficacy comparison of the machine learning models

**Figure 5:**
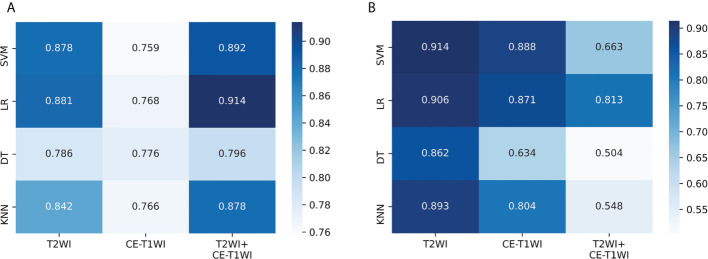
Performance of twelve machine learning models on the two test sets. **(A)** The AUC values in the test set 1. **(B)** The AUC values in the test set 2. SVM, support vector machine; LR, logistic regression; DT, decision tree; KNN, k-nearest neighbor; T2WI, T2-weighted image; CE-T1WI, contrast–enhanced T1-weighted image; T2WI+CE-T1WI, the combination of T2-weighted image and contrast–enhanced T1-weighted image.

The predictive efficacies of twelve different machine learning models are summarized in **Table 4**. According to results, the best performing model used only T2WI sequence and achieved highest AUC of 0.914 (95%CI: 0.806-0.973) in the test set 2. The model derived from combined sequence (T2WI+CE-T1WI) had comparable performance to the T2WI sequence model in the training set and test set 1, but its predictive performance decreased in the test set 2. Meanwhile, four different classifiers exhibit different performances. Compared with the other three classifiers, SVM showed the highest predictive efficacy on the two test sets (AUC= 0.878 and 0.914, respectively). The classification performance of LR was comparable to that of SVM (AUC= 0.881 and 0.906, respectively). The models trained with DT classifier showed the worst performance in all test sets (AUC= 0.786 and 0.862, respectively). The AUC values of twelve machine learning models on the two test sets were visualized as a heatmap (**Figure 5**). To sum up, the T2WI-SVM model demonstrated relatively stable and optimal predictive efficacy in all machine learning models for classification tasks on the two test sets, and served as the optimal radiomics model.

**Table 4:** Predictive efficacies of different machine learning models.

Model		SVM			LR			DT			KNN		
		Train	Test 1	Test 2	Train	Test 1	Test 2	Train	Test 1	Test 2	Train	Test 1	Test 2
T2WI	AUC	0.901	0.878	0.914	0.916	0.881	0.906	0.954	0.786	0.862	0.924	0.842	0.893
	95%CI	[0.837-0.946]	[0.765-0.949]	[0.806-0.973]	[0.856-0.957]	[0.769-0.951]	[0.795-0.968]	[0.904-0.983]	[0.659-0.883]	[0.741-0.941]	[0.866-0.963]	[0.722-0.924]	[0.779-0.941]
	Accuracy	0.815	0.793	0.833	0.867	0.828	0.833	0.867	0.776	0.815	0.830	0.810	0.852
	Sensitivity	0.831	0.788	0.935	0.870	0.818	0.871	0.870	0.788	0.806	0.818	0.758	0.935
	Specificity	0.793	0.800	0.696	0.862	0.840	0.783	0.862	0.760	0.826	0.845	0.880	0.739
CE-T1WI	AUC	0.865	0.759	0.888	0.863	0.768	0.871	0.951	0.776	0.634	0.894	0.766	0.804
	95%CI	[0.796-0.918]	[0.628-0.861]	[0.772-0.957]	[0.793-0.916]	[0.639-0.869]	[0.752-0.947]	[0.900-0.981]	[0.648-0.875]	[0.492-0.761]	[0.830-0.941]	[0.636-0.867]	[0.673-0.899]
	Accuracy	0.770	0.724	0.852	0.748	0.741	0.815	0.881	0.707	0.704	0.800	0.672	0.778
	Sensitivity	0.805	0.727	0.903	0.792	0.788	0.871	0.883	0.667	0.903	0.779	0.697	0.774
	Specificity	0.724	0.720	0.783	0.690	0.680	0.739	0.879	0.760	0.435	0.828	0.640	0.783
T2WI+ CE-T1WI	AUC	0.931	0.892	0.663	0.946	0.914	0.813	0.967	0.796	0.504	0.944	0.878	0.548
	95%CI	[0.874-0.967]	[0.783-0.958]	[0.522-0.786]	[0.893-0.977]	[0.810-0.971]	[0.684-0.906]	[0.922-0.990]	[0.669-0.890]	[0.365-0.643]	[0.890-0.976]	[0.765-0.949]	[0.407-0.684]
	Accuracy	0.852	0.793	0.685	0.874	0.810	0.741	0.896	0.759	0.648	0.837	0.810	0.556
	Sensitivity	0.831	0.758	0.806	0.857	0.788	0.806	0.922	0.758	0.774	0.805	0.727	0.516
	Specificity	0.879	0.840	0.522	0.897	0.840	0.652	0.862	0.760	0.478	0.879	0.920	0.609

T2WI, T2-weighted image; CE-T1WI, contrast–enhanced T1-weighted image; T2WI+CE-T1WI, the combination of T2-weighted image and contrast–enhanced T1-weighted image; AUC, area under the curve; CI, confidence interval; SVM, support vector machine; LR, logistic regression; DT, decision tree; KNN, k-nearest neighbor.

### Performance assessment of the clinical model, radiomics model and combined model

**Figure 6:**
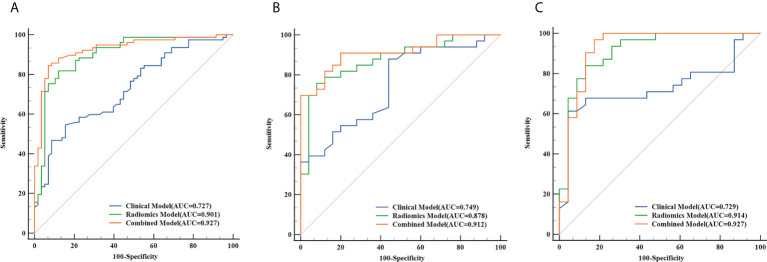
The receiver operating characteristic (ROC) curves of the clinical model, the radiomics model (T2WI+SVM) and the combined model in the training set **(A)**, test set 1 **(B)** and test set 2 **(C)**, respectively.

**Figure 7:**
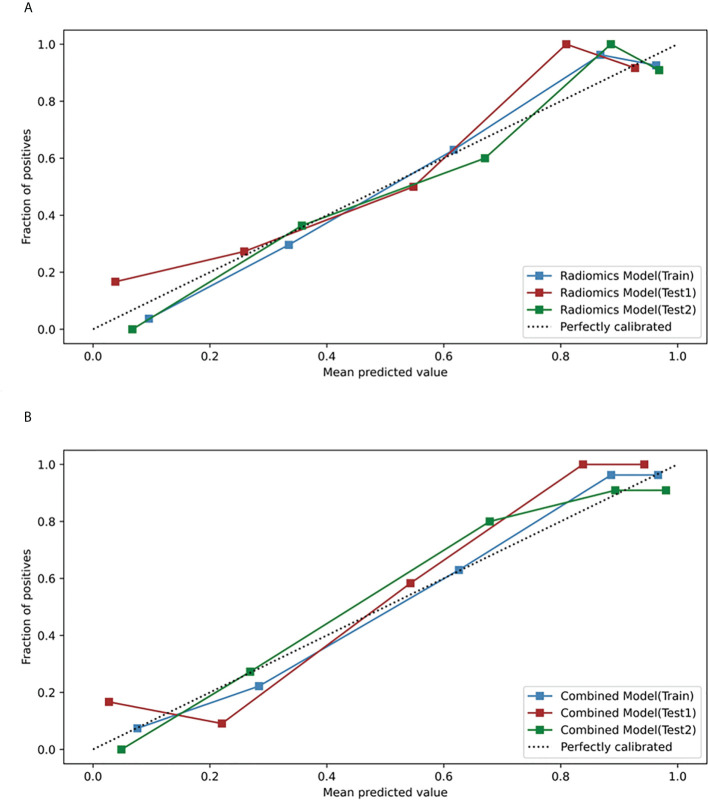
Calibration curves of the radiomics model and the combined model in the training, test 1 and test 2 sets. **(A)** Calibration curves of the radiomics model (T2WI+SVM). **(B)** Calibration curves of the combined model.

Based on the five significant clinic-radiological features selected by univariate analysis, SVM algorithm was used to build the clinical model. Meanwhile, the combined model was constructed with the combination of the best radiomics feature subsets (T2WI) and five significant clinic-radiological features by using SVM algorithm. **Table 5** reports the diagnostic capacity of the clinical model, the radiomics model (T2WI+SVM) and the combined model, the corresponding ROC curves are presented in **Figure 6**. As it turns out, the combined model achieved highest AUC of 0.912 (95%CI: 0.807-0.970) and 0.927(95%CI: 0.823-0.980) for differentiation of SNIP and MST in test 1 and test 2 sets, which performed prominently better than clinical model (*P*=0.011 and *P*=0.005, respectively), but not significantly different from the radiomics model (*P*=0.100 and *P*=0.452, respectively). Next, the radiomics model (T2WI+SVM) yielded AUC of 0.878 (95%CI: 0.765-0.949) and 0.914 (95%CI: 0.806-0.973) in two test sets, which significantly outperformed the clinical model in the test set 2 (*P*=0.011), but no significant difference was found in the test set 1 (P=0.064). Finally, the clinical model showed a relatively poor diagnostic performance in all sets, with the AUCs of 0.727 (95%CI: 0.644-0.800), 0.749 (95%CI: 0.618-0.854) and 0.729 (95%CI: 0.591-0.841) in the training, test 1 and test 2 sets, respectively. The calibration curves demonstrated good agreement between predicted and actually observed of the radiomics model (T2WI+SVM) and the combined model (**Figure 7**).

**Table 5:** Diagnostic performance of the clinical model, the radiomics model and the combined model.

Group	Model	AUC (95%CI)	Accuracy	Sensitivity	Specificity	NPV	PPV	*P*-value
Training set	Clinical model	0.727 [0.644-0.800]	0.674	0.831	0.466	0.675	0.674	*P* ^a^ *<*0.001
	Radiomics model (T2WI+SVM)	0.901 [0.837-0.946]	0.815	0.831	0.793	0.780	0.842	*P* ^b^ =0.062
	Combined model	0.927 [0.870-0.965]	0.874	0.883	0.862	0.847	0.895	*P* ^c^ *<*0.001
Test set 1	Clinical model	0.749 [0.618-0.854]	0.724	0.879	0.520	0.765	0.707	*P* ^a^ =0.064
	Radiomics model (T2WI+SVM)	0.878 [0.765-0.949]	0.793	0.788	0.800	0.741	0.839	*P* ^b^ =0.100
	Combined model	0.912 [0.807-0.970]	0.845	0.818	0.880	0.786	0.900	*P* ^c^ =0.011
Test set 2	Clinical model	0.729 [0.591-0.841]	0.648	0.710	0.565	0.591	0.688	*P* ^a^ =0.011
	Radiomics model (T2WI+SVM)	0.914 [0.806-0.973]	0.833	0.935	0.696	0.889	0.806	*P* ^b^ =0.452
	Combined model	0.927 [0.823-0.980]	0.870	0.871	0.870	0.833	0.900	*P* ^c^ =0.005

The P-value was calculated by the Delong test. P^a^ means the Delong test between the clinical model and the radiomics model; P^b^ means the Delong test between the radiomics model and the combined model; P^c^ means the Delong test between the clinical model and the combined model. AUC, area under the curve; CI, confidence interval; PPV, positive predictive value; NPV, negative predictive value.

## Discussion

We compared the classification performance of twelve different machine learning models composed of single-sequence, combined-sequence and four classifiers, and determined that T2WI-SVM classifiers served as the best performing machine learning model for differential diagnosis of SNIP and MST. Moreover, the combination of radiomics features and clinic-radiological features significantly improved the diagnostic performance of model, compared to the clinical model alone (*P*=0.011, 0.005). The good performance and robustness of our final models were validated by test set 1 as well as an additional independent test set 2 with different scanners.

Conventional radiology emphasized an active role of morphological characteristics in qualitative diagnosis (3, 22). Our results indicated that CCP sign, tumor heterogeneity, adjacent bone involvement and infiltration of surrounding tissue were significant morphological features in differentiation between SNIP and MST. Signs of extensive osseous lytic destruction and significant surrounding soft tissue infiltrate strongly suggest malignancy tumor, while localized bone sclerosis and inhomogeneous signal intensity caused by convoluted cerebriform pattern signs are characteristic features of benign SNIP. The convoluted cerebriform pattern was revealed to be a valuable sign for differential diagnosis, and actually reflected the histological architecture of the SNIP (the hyperplastic epithelium grows into the underlying stroma) (1, 23). Our results are consistent with previous studies (4, 8, 24). Unfortunately, the clinical model exhibited a relatively poor predictive performance for classifying SNIP and MST. Even though traditional radiology diagnostic methods are convenient and cost effective in routine clinical practice, the clinic-radiological features alone are not sufficient to accurately differentiate SNIP from MST in a substantial number of cases.

Until lately, the performance of radiomics analysis has been compared to that of practicing radiologists in the differential diagnosis of sinonasal tumors. In the Ramkumar et al. study (15), texture analysis was applied to differentiate 22 SNIPs from 24 MSTs, the accuracy achieved by texture analysis (89.1%) was significantly better than that of the ROI-based neuroradiologists’ review (56.5%, P=0.0004). However, small sample size reduces the reliability of the results, and simple texture analysis might not be sufficient to capture more valuable higher-order features and also unable to combine clinical data for comprehensive analysis. Zhang et al. (17) reported that margin, bone involvement, and rad-score were independent indicators of sinonasal tumor malignancy, the radiomic nomogram based on pre-contrast MRI image achieved the AUC of 0.91, performed significantly better than that of the clinical model (AUC=0.83, P<0.001) in predicting malignant sinonasal tumors. However, in Zhang’s study, only pre-contrast MR images were used for features extraction, which has potential to miss some important information related to tumor angiogenesis and vascular permeability.

Choosing the most appropriate machine learning methods according to different classification objects is one of the critical issues in radiomics research. In contrast to previous studies (15, 17), we developed more machine learning models by combining different classifiers and sequences. Notably, our research indicated that SVM classifiers did better than other three classifiers in clinical classification tasks of sinonasal tumors. SVM is a supervised machine learning algorithm, which has been widely used for various classification and regression tasks (25). The problems of latitude disaster and overfitting of the small specimen model can be handled well by SVM, and the model trained with it has high generalization ability and prediction accuracy (26). In addition, the selection of MRI sequence is one of the factors affecting the performance of the model. In our study, models based on T2WI sequence achieved good performance on two test sets. The combined sequence model (T2WI+CE-T1WI) had comparable performance to the T2WI sequence model in the training set and test set 1, but its predictive performance significantly decreased in the test set 2. This may indicate that the generalization performance of the combined sequence model is not as good as that of the single sequence model. We considered that this may be related to the feature selection method and the fact that our models were only trained on the single-scanner training set.

Radiomics features are related to the intra-tumoral heterogeneity, and may help explain the complex tumor biological behavior (13, 14). Among the radiomics features extracted in our study, small area emphasis (SAE), IDM and maximal correlation coefficient (MCC) can be served as indicators to quantify the intensity heterogeneity and texture complexity of tumor (27). SAE is a measure of the distribution of small size areas, with a higher value indicates a smaller area and finer texture. IDM (also known as Homogeneity 2) and MCC are the measures of the local homogeneity and texture complexity of the image, respectively. These features emphasizes that MST are more heterogeneous and complex in spatial texture than SNIP. Pathological evidences reflect that histological inner component of MST tend to be more complicated and disordered in terms of cell proliferation, arrangement and angiogenesis than benign SNIP (3, 28, 29). This subtle histological difference could be barely recognized through the naked eye, but detected by radiomics.

Compared with traditional morphological features, radiomics features has the potential to provide more comprehensive, quantitative tumor heterogeneity information, which can be helpful in interpreting the potential relationship between pathophysiological properties and radiology imaging phenotype. Furthermore, the complementarity between radiomics features and clinic-radiological features has been demonstrated in this study, the combination of both features could improve the performance of model.

There are several limitations of the current study. First, our models were trained on the single-scanner training set, which might degrade the generalization performance of the models. Although the good performance of our models was verified by an independent test set 2 with different scanners, the small sample size may result in biased results. Our group plans to conduct a cross-institution collaboration trial to expand the sample size and carry out further cross-validation. Second, complex anatomical structure and ill-defined margin bring barriers to the manual segmentation of sinonasal neoplasm, which makes the segmentation process time-consuming and complicated. Semi-automatic or automatic segmentation methods (30) can be explored to improve the efficiency and accuracy of lesion segmentation. Third, the current binary classification radiomics research is not sufficient to meet the complicated clinical diagnosis needs, deep learning (31) is expected to achieve the multi-class classification task, simplify the process and capture more high-level features. Last but not the least, only conventional MRI images were used for radiomics analysis in this study. It has been reported that functional MRI (such as DWI, ADC and DCE-MRI) can provide additional valuable information for the discrimination between SNIP and MST (9, 10), but we did not include functional images due to limited sample size and insufficient image quality. Future work needs exploring the effective role of functional MRI in radiomics research. Anyhow, when radiomics is applied in clinical practice, more rigorous studies with multicenter and large-scale data set are needed to supply more evidence to confirm its feasibility.

## Conclusion

In conclusion, we developed different machine learning models for preoperative distinction of SNIP and MST, and demonstrated that T2WI-SVM classifier achieved best predictive efficacy in clinical classification tasks. Moreover, the incorporation of radiomics features and clinic-radiological features helped improve model performance. In other words, radiomics integrated with clinic-radiological features has potential to provide a non-invasive and credible predictions to clinicians, which may facilitate better clinical decision making.

## Data availability statement

The original contributions presented in the study are included in the article/**Supplementary Material**. Further inquiries can be directed to the corresponding author.

## Ethics statement

The studies involving human participants were reviewed and approved by the Institutional Review Board of the First Affiliated Hospital of Chongqing Medical University. Written informed consent from the participants’ legal guardian/next of kin was not required to participate in this study in accordance with the national legislation and the institutional requirements.

## Author contributions

JP, JG, and QY designed the research and drafted the manuscript. JG, QY, QL, and BG collected the data and preprocessed data. JG, QY, and XZ performed major data analyses. JP and FL participated in the review and editing. All authors contributed to the article and approved the submitted version.

## Funding

This project received support from The Foundation of Science and Technology Bureau of Yuzhong District, Chongqing, China (Grant No. 20190111) and the Natural Science Foundation of Chongqing, China (Grant No. cstc2021jcyj-msxmX0020).

## Conflict of interest

The authors declare that the research was conducted in the absence of any commercial or financial relationships that could be construed as a potential conflict of interest.

## Publisher’s note

All claims expressed in this article are solely those of the authors and do not necessarily represent those of their affiliated organizations, or those of the publisher, the editors and the reviewers. Any product that may be evaluated in this article, or claim that may be made by its manufacturer, is not guaranteed or endorsed by the publisher.
