# Supplementary material for: MRI radiomics-based machine learning model integrated with clinic-radiological features for preoperative differentiation of sinonasal inverted papilloma and malignant sinonasal tumors
Source: Front Oncol. 2022 Sep 23;12:1003639. doi: 10.3389/fonc.2022.1003639 (PMC9538572; doi:10.3389/fonc.2022.1003639)
Supplement: Supplementary file 1 [file Table_1.docx]

Supplementary Material

**Supplementary TABLE S1 |** Detailed MRI Scan Parameters.

| **MRI Scanner** | **Scanner A** | | **Scanner B** | | **Scanner C** | |
| --- | --- | --- | --- | --- | --- | --- |
|  | Siemens Essenza 1.5T | | GE Signa HDxt 3.0T | | Siemens Skyra 3.0T | |
| Sequence | T1WI | T2WI | T1WI | T2WI | T1WI | T2WI |
| TR (ms) | 769 | 4260 | 400 | 3780 | 644 | 3000 |
| TE (ms) | 10 | 86 | 12 | 85 | 10 | 93 |
| FOV (cm^2^) | 22×22 | 22×22 | 22×22 | 22×22 | 24×24 | 24×24 |
| NEX | 2 | 2 | 2 | 2 | 2 | 2 |
| Acquisition matrix | 256×204 | 256×204 | 288×224 | 288×224 | 320×224 | 320×224 |
| Slice thickness (mm) | 5 | 5 | 4 | 4 | 4 | 4 |
| Slice gap (mm) | 1 | 1 | 1 | 1 | 1 | 1 |

TR, repetition time; TE, echo time; FOV, field of view; NEX, number of excitations.

**Supplementary TABLE S2** | The consistency test for the MRI radiological features evaluation between two radiologists.

| **MRI radiological features** | **Kappa** | ***P* value** |
| --- | --- | --- |
| Location | 1.000 | ＜0.001 |
| Extent | 1.000 | ＜0.001 |
| Shape | 0.886 | ＜0.001 |
| Margin | 0.862 | ＜0.001 |
| Convoluted cerebriform pattern sign | 0.818 | ＜0.001 |
| T1 high signal | 0.933 | ＜0.001 |
| T2 low signal | 0.873 | ＜0.001 |
| Heterogeneity | 0.856 | ＜0.001 |
| Necrosis | 0.885 | ＜0.001 |
| Adjacent bone involvement | 0.828 | ＜0.001 |
| Infiltration of surrounding tissue | 0.816 | ＜0.001 |
